# Guy's Hospital

**Published:** 1829-01-01

**Authors:** 


### GUY'S HOSPITAL.

### I. Injection of the Cavity of a Psoas Abscess.

The province of surgery appears to be undergoing a total revolution in the BOROUGH. Some little time ago they hit upon the plan of curing scirrhus of the testis by tying, or excising its excretory duct, and now they are treating psoas abscess by injection! Time was when the surgeon was constantly in dread of admitting even air into the cavity of this abscess—when each had his plan of preventing the ill effects, believed, or supposed to follow its mere *opening*. Mr. Abernethy carefully squeezed out the contents, and instantly closed the puncture by adhesive plaster, whilst others abstained from employing the least pressure, but contented themselves with allowing the contents to *drain* in a poultice. These various precautions, these obvious fears, argue the existence of some degree of danger, real or imaginary, and lead us to regard with feelings of surprise, we might almost say, dismay, the practice pursued in the following case.

John Cogle, a scrofulous young man, was admitted into Guy's Hospital on the 21st of May, and placed under the care of Mr. Key.

On the inside of the thigh was a tumour of considerable size, dilatable on coughing, the integuments over which were thin, at one part red and inflamed. The patient was pale, emaciated, and hectic; the bowels were regular; the appetite bad; the constitutional irritation and disturbance considerable. The disease would appear to have originated in a strain received in the back more than a year before admission; the tumour in the groin was of ten or eleven months standing. He was put on a generous diet, and ordered a grain of the sulphate of quinine, eight drops of dilute sulphuric acid, and two ounces of infusion of roses, twice daily. Wine and porter were likewise allowed. In the early part of June, the abscess had nearly closed, its walls were thin and flaccid, and the pus which it contained was diminished in quantity. The bowels were costive, the general symptoms but little relieved.

"Mr. Key wished that what pus was left in the cyst should be evacuated, and that a lotion, composed of two grains of the sulphate of zinc to two ounces of water, should be injected once a day into the cyst. The limb to be bandaged with a roller carried up to the groin, in order to produce, if possible, cohesion of the sides of the cavity. The patient was ordered to take two grains of the sulphate of quinine, half a drachm of the sulphate of magnesia, 30 drops of tincture of henbane, and one ounce of water, twice daily." As a natural consequence, this injecting produced an accession of fever, and was necessarily discontinued, whilst, to calm the excitement, effervescing mixtures were required. On the 26th of June, the sides of the abscess had in great measure cohered, and the patient appeared to be better. The injection was a second time tried, and appeared to produce no obvious ill effect. However, he emaciated even more rapidly than ever, and, early in July, a fluctuating tumour made its appearance in the loins, on the *opposite* side. The hectic was now severe, and on opening this abscess a pint and a half of matter was discharged. A piece of lint was inserted in the opening to prevent its closing, and a linseed poultice applied! The patient lingered on till the 4th of September, when he sank. No inspection of the body was allowed by the friends.

We really hope, for the sake of the surgeon, that the above report may be found to be inaccurate, for of all the modes of treating psoas abscess which ever we heard of, this appears the most extraordinary. We never saw a case of the disease, where it was not connected with disease of the bones or intervertebral substance of the spine, whilst the swelling which appeared in the opposite loin proves to demonstration, that such complication obtained in the present instance. Knowing that this is almost always the case, that the abscess may be called constitutional, generally occurring in, and ever an augur of a bad and a scrofulous habit; bearing in mind that it *points* in the thigh, but *arises* in the back, what can we expect from the closure, by art, of its external opening? The sequel of this case is an answer to the question; a larger and more formidable collection of matter in the loins, in consequence of the discharge being denied a ready exit below! Another point in the treatment is a matter of curiosity. In the *lower* abscess, Mr. Key used injection and strapping in order to produce adhesion of the walls, whilst the *upper* was treated in a manner diametrically opposite, viz. by the introduction of a tent to maintain the opening! This is surely blowing hot and cold!

### II. COMPOUND FRACTURE OF THE TIBIA AND FIBULA—DIFFICULTY OF REDUCTION—AMPUTATION.

A stout-looking man, who had abstemiously confined himself to the copious imbibition of porter and ale, received from an apparently trifling injury, a compound fracture of the tibia and fibula, the wound being small, and the bone not protruding. The reduction was accomplished with considerable difficulty; the limb then bound up for some inches above and below the fracture with alternate straps of adhesive plaster; a splint applied on each side from the ankle to knee; the patient laid supine; and "the leg placed on the bed in a fracture-box, of such a height, that the leg alone was raised about eight inches from the bed; both thigh and leg being consequently semiflexed;" a description which we cannot at all comprehend, unless it is *figurative* of a double-inclined plane. This was on the 5th July, and on the evening of the 7th, re-action having taken place, the limb was become hot and painful, and swelling had come upon the ankle and lower part of the leg. Several of the straps were cut through with relief—pulse 110, full and strong—tongue white and coated—bowels confined. Three grains of the submuriate, and six of the compound extract of colocynth were directed to be taken, but although the limb was less painful on the 7th, a number of vesicles had appeared around the fracture, and the pulse was rather smaller and quicker.

On the 8th, the limb was exceedingly painful, the tongue less furred, pulse 100, and jerky. The straps were now removed, but a roller remained above and below. The bones were as yet in apposition, the wound appeared sloughy, and next day presented a slough an inch square. The integuments around were dark-coloured, and covered with bullæ, the discharge very fetid and profuse, pulse 120, tongue cleaner. The uppermost bandage was now cut away, and the leg appeared straight. On the 10th, all the symptoms were worse, the limb still more swollen, the skin darker coloured. *Calomel and opium at bed time.*

The severer symptoms now began to fade, but the bones became displaced, the upper portion of the tibia projecting through the wound. The first apparatus was now removed, powerful extension put in force, apposition obtained, and the limb laid out straight, the heel being raised a few inches. A splint was lightly fastened on each side the limb by means of tapes, and a poultice laid over the wound. On the very next day, the protrusion of the upper part of the tibia reappeared, and continued in spite of every possible change of position. A part of the tibia was even sawed off without effect, which obliged Mr. Key to resort to amputation after all. On dissecting the limb, it was found that the tibia was fractured obliquely, whilst a splinter of bone very nearly detached, had transfixed the flexor longus digitorum, proving, thereby, a continual source of irritation to the muscle, and stimulating it constantly to contraction. This contraction, aided by the inflammation, of necessity set up, and preventing the muscle from duly relaxing, inevitably dragged up the lower part of the tibia, and forced the extremity of the upper through the wound.—*Gazette.*

This is the explanation offered by the reporter, and it may be, nay probably is, the correct one. We have heard it remarked, by a person well qualified to give an opinion on the subject, that fractures, in general, are not managed well in the hospitals of the Borough. Without giving in to so sweeping a charge, we are "free to confess" that the practice pursued in this particular case, is not altogether to our taste. A full-bodied man, accustomed to the use, we should rather say *abuse*, of malt liquors, receives a compound fracture of the leg, without the infliction of any great shock on the system at large. The first thing that is done is to *strap* up the leg, in other words, apply the most unyielding compression to a part which must shortly and inevitably swell. This we do not like. In the next place, re-action is established; the limb is hot and painful, the tongue white and coated, the pulse above a hundred, full and strong. Surely venesection in such a state of things is required; surely the exhibition of a purgative pill is little more than milk and water practice.* We really might remark in a similar tone on several other items in the treatment of this case, but shall merely observe, that a want of activity seems to have pervaded the whole. We hate picking critical holes in the literary coats of other people, but it is a duty from which we dare not shrink, to comment on error whenever we think (it may be erroneously on our own part) that it obtains.

* The double-inclined plane was adopted. In fractures of the leg we think the straight position is generally preferable. Nothing is more easily managed than a "junk."

### XLVIII.

### GUY'S HOSPITAL.

FRACTURES.—The difficulty attending the diagnosis of fractures in and about the neck of the thigh-bone, is notorious. The following case is a curious instance of the absence of symptoms indicative of the injury. It is taken from Mr. Amesbury's recent and very useful work.

Mrs. Shaftoe, aged 62, fell, on London Bridge, and pitched upon the stones, in the carriage-way. She was picked up, and walked, with assistance, a distance of about three hundred yards, not without considerable pain and limping. From the top of St. Thomas's Street, to the gates leading into Guy's Hospital, she walked *without assistance*, but here she felt her limb give way, and it was necessary to carry her from this to her bed. Mr. Key, who immediately saw her, discovered no fracture, and next day Sir Astley Cooper examined the limb with the same results. She could bend the limb upon the pelvis, and although the limb became slightly everted when left to itself, she was able to roll it inward, "so as to bring it to what might be called its *supine* position."* The whole of the inconvenience was referred by the patient to the upper and inner part of the thigh, and the hollow behind the trochanter major. Seventeen days afterwards, Mr. Amesbury examined the limb, and by rolling it inwards and bending it upon the pelvis, found that the bone was fractured through the neck. Sir Astley Cooper, next day, repeated the examination, and corroborated Mr. Amesbury's opinion, though he thought the fracture was external to the capsule. The common fracture-box was now applied, but removed on account of the inconvenience it occasioned, and Mr. A.'s apparatus substituted in its stead.

"Before the apparatus was applied, the limb was lying in the extended position, and retracted about an inch. The foot was everted. Crepitus could easily be produced. The limb could be brought down to its proper length with facility by extension, but it was again retracted as soon as the extending force was discontinued. The apparatus was worn, with the limb in the straight position, from the 17th Jan. to the 12th of the following March. Its use was now discontinued, as I considered the fracture was united, though not without shortening of the limb."*

Mr. Amesbury believes, that in cases like these, when the patient can support himself for a time upon the limb, and the symptoms are at first so obscure, the neck is broken off at its junction with the shaft, and driven into the cancellated structure of the great trochanter, remaining there firmly locked for a greater or shorter time. The mode in which such accidents occur is, by falls on the great trochanter, or a force which will operate in a similar direction.

"The symptoms of fracture of the neck of the bone thus situated, are not such as will enable us to decide, with certainty, in all cases, upon what part of the cervix is broken. More or less shortening of the limb, pain at the upper and inner part of the thigh, and behind the trochanter, more or less tumefaction soon after the accident, great diminution of power in the limb, and absolute lameness, are the only symptoms I have noticed, till the neck of the bone is disengaged from the trochanter, so as to move independently of it; and then the crepitus of fracture might, also, commonly be felt when the limb is rotated or bent upon the pelvis."

Other causes will, also, produce the phenomenon of the patient being able to walk, with some assistance, immediately after the accident, for example, the absence of laceration in the neighbouring textures, or an incomplete fracture.

---

* We should say, its *prone* position. It *might* be called supine, no doubt, but then it would be entirely wrong.

* "This woman lived several years after the accident; and was able to walk about with facility, without a stick or any other assistance, though not without limping, in consequence of the shortened state of the limb. After her death, Sir Astley Cooper obtained the upper part of the thigh-bone; and, he has stated to me, in conversation, that the bone is consolidated, that the cervix is absorbed, and, that there is no appearance of callus. From the last observation, I infer, that the callus has become cancellated. I have not learned at what part of the cervix the fracture appeared upon inspection to have taken place."

